# Cryptorchidism Is Frequently Associated With Testicular Dysfunction

**DOI:** 10.1210/jendso/bvae025

**Published:** 2024-02-12

**Authors:** Rodolfo A Rey

**Affiliations:** Centro de Investigaciones Endocrinológicas “Dr. César Bergadá” (CEDIE), CONICET—FEI—División de Endocrinología, Hospital de Niños Ricardo Gutiérrez, C1425EFD Buenos Aires, Argentina; Universidad de Buenos Aires, Facultad de Medicina, Departamento de Histología, Embriología, Biología Celular y Genética, C1121ABG Buenos Aires, Argentina; Instituto de Investigaciones Biomédicas, S3000FNF Santa Fe, Argentina

**Keywords:** AMH, cryptorchidism, DSD, FSH, germ cells, inhibin B, Leydig cells, Sertoli cells, testosterone, undescended testes

Cryptorchidism or undescended testis, that is, testis that is not located at or below mid-scrotum, is one of the most common congenital abnormalities in newborns, with a prevalence between 1.6% and 9.0%, according to different series [1]. Additionally, acquired cryptorchidism, or *ascensus testis*, refers to postnatal testicular ascent occurring when the cremasteric reflex is established [1]. In sum, cryptorchidism is a frequent reason for seeking medical attention in pediatric age.

The gonads originate near the kidneys during the first trimester of intrauterine life and descend to the scrotum following a biphasic model. The 3 hormones produced by the fetal testis, testosterone, insulin-like peptide 3 (INSL3) and anti-Müllerian hormone (AMH), have been suggested to promote testis descent. In the initial or transabdominal phase of testicular descent, INSL3 is involved in the thickening of the gubernaculum, whereas testosterone provokes the regression of the cranial ligament and swelling of the gubernaculum. In the second or inguino-scrotal phase, the testis descends along the inguinal canal into the scrotum, induced by androgens and by the increase in intraabdominal pressure as the muscular abdominal walls develop. AMH has been suggested to participate in testicular descent, although its precise action remains unclear [2]. Cryptorchidism may be the consequence of a primary testicular dysfunction, of a central disorder affecting the gonadotropin-releasing hormone (GnRH)-gonadotroph axis, or of an anatomic malformation independent of any endocrine disorder.

Surprisingly, for decades, the management of boys with cryptorchidism has focused on bringing the testes to the scrotum in an adequate and timely manner, as if placing the gonads in their proper position solved everything, without much attention having been paid to their functional status. Indeed, most clinical guidelines have been produced by scientific societies of pediatric urology with little participation of specialists in pediatric endocrinology [3, 4]. However, it seems clear that assessing the function of the hypothalamic-pituitary-testicular axis at the time of diagnosis can help to understand the pathophysiology of cryptorchidism in each patient and its potential fertility prognosis, as well as to decide which is the most adequate treatment and appraise its effect [5, 6]. For instance, orchiopexy may not be the first option in boys with a history compatible with hypogonadotropic hypogonadism, reflected in the absence of the activation of the hypothalamic-pituitary-testicular axis normally occurring during the first 3 to 6 months after birth (called *minipuberty* by many authors). Indeed, therapy with gonadotropins has shown high efficacy in provoking testicular descent in infants with congenital hypogonadotropic hypogonadism [7].

The recent study by Lucas-Herald and colleagues [8] analyzed the prevalence of endocrine abnormalities in a large series of patients undergoing bilateral orchiopexy in a tertiary pediatric center in the past 15 years. Supporting the notion that testicular function is neglected at diagnosis, only 53% of the 243 cases had been referred to the endocrine team before surgical treatment. Hormone assessment included serum AMH determination in 87 boys, testosterone measurement after human chorionic gonadotropin (hCG) stimulation in 82, and gonadotropin assay following a GnRH test in 60 cases, at a median age of 1 year. Interestingly, evidence of testis dysfunction was observed in 38% of the boys: basal serum AMH was low and testosterone response to hCG was insufficient in 31%, while follicle-stimulating hormone (FSH) and luteinizing hormone (LH) were elevated in respectively 22% and 41% of the cases after GnRH stimulation. Furthermore, next-generation sequencing assessment revealed the existence of a genetic variant in 18% of the boys with isolated bilateral undescended testes and up to 41% in those with complex cryptorchidism, that is, with additional genital features such as micropenis or hypospadias.

The results of this large study in a tertiary center in the United Kingdom highlights the need for developing a systematic approach to the investigation of boys with undescended testes before treatment is decided. Although a proportion of the patients of this large series were assessed for testis function after orchiopexy, a similarly relevant frequency of testicular dysfunction has been observed in other series of boys with cryptorchidism before surgical treatment [5, 6]. In conclusion, evidence of hypogonadism and genetic findings are common features in boys presenting with cryptorchidism, and endocrine and genetic assessment should be considered as routine clinical management.

## Disclosures

The author has nothing to disclose.

## Abbreviations

AMH: anti-Müllerian hormone
FSH: follicle-stimulating hormone
GnRH: gonadotropin-releasing hormone
hCG: human chorionic gonadotropin
INSL3: insulin-like peptide 3

## References

[1] Virtanen HE, Bjerknes R, Cortes D, et al Cryptorchidism: classification, prevalence and long-term consequences. Acta Paediatr. 2007;96(5):611‐616. 17462053 10.1111/j.1651-2227.2007.00241.x

[2] Hutson JM, Balic A, Nation T, Southwell B. Cryptorchidism. Semin Pediatr Surg. 2010;19(3):215‐224. 20610195 10.1053/j.sempedsurg.2010.04.001

[3] Kolon TF, Herndon CD, Baker LA, et al Evaluation and treatment of cryptorchidism: AUA guideline. J Urol. 2014;192(2):337‐345. 24857650 10.1016/j.juro.2014.05.005

[4] Radmayr C, Dogan HS, Hoebeke P, et al Management of undescended testes: European Association of Urology/European Society for Paediatric Urology Guidelines. J Pediatr Urol. 2016;12(6):335‐343. 27687532 10.1016/j.jpurol.2016.07.014

[5] Verkauskas G, Malcius D, Eidukaite A, et al Prospective study of histological and endocrine parameters of gonadal function in boys with cryptorchidism. J Pediatr Urol. 2016;12(4):238.e1‐238.e6. 10.1016/j.jpurol.2016.05.007 27321556

[6] Grinspon RP, Gottlieb S, Bedecarras P, Rey RA. Anti-Müllerian hormone and testicular function in prepubertal boys with cryptorchidism. Front Endocrinol (Lausanne). 2018;9182:181‐114. 10.3389/fendo.2018.00182 PMC5996917 29922225

[7] Lambert AS, Bougnères P. Growth and descent of the testes in infants with hypogonadotropic hypogonadism receiving subcutaneous gonadotropin infusion. Int J Pediatr Endocrinol. 2016;2016:13. 27379168 10.1186/s13633-016-0031-9 PMC4931699

[8] Lucas-Herald AK, Alkanhal KI, Caney E, et al Gonadal function in boys with bilateral undescended testes. J Endocr Soc. 2024;8(2):bvad153. 38205164 10.1210/jendso/bvad153 PMC10777671

